# Prehospital Notification Procedure Improves Stroke Outcome by Shortening Onset to Needle Time in Chinese Urban Area

**DOI:** 10.14336/AD.2017.0601

**Published:** 2018-06-01

**Authors:** Sheng Zhang, Jungen Zhang, Meixia Zhang, Genlong Zhong, Zhicai Chen, Longting Lin, Min Lou

**Affiliations:** ^1^Department of Neurology, the Second Affiliated Hospital of Zhejiang University, School of Medicine, Hangzhou, China; ^2^Hangzhou Emergency Medical Center of Zhejiang Province, Hangzhou, China; ^3^The School of Medicine and Public Health, University of Newcastle, Newcastle, Australia

**Keywords:** thrombolysis, prehospital notification, emergency medical service, onset to needle time, door to needle time, clinical outcome

## Abstract

Intravenous thrombolysis (IVT) with recombinant tissue plasminogen activator (rt-PA) can improve clinical outcome in eligible patients with acute ischemic stroke (AIS). However, its efficacy is strongly time-dependent. This study was aimed to examine whether prehospital notification by emergency medical service (EMS) providers could reduce onset to needle time (ONT) and improve neurological outcome in AIS patients who received IVT. We prospectively collected the consecutive clinical and time data of AIS patients who received IVT during one year after the initiation of prehospital notification procedure (PNP). Patients were divided into three groups, including patients that transferred by EMS with and without PNP and other means of transportation (non-EMS). We then compared the effect of EMS with PNP and EMS use only on ONT, and the subsequent neurological outcome. Good outcome was defined as modified Rankin Scale score of 0-2 at 3-months. In 182 patients included in this study, 77 (42.3%) patients were transferred by EMS, of whom 41 (53.2%) patients entered PNP. Compared with non-EMS group, EMS without PNP group greatly shortened the onset to door time (ODT), but EMS with PNP group showed both a significantly shorter DNT (41.3 ± 10.7 min vs 51.9±23.8 min, t=2.583, p=0.012) and ODT (133.2 ± 90.2 min vs 174.8 ± 105.1 min, t=2.228, p=0.027) than non-EMS group. Multivariate analysis showed that the use of EMS with PNP (OR=2.613, p=0.036), but not EMS (OR=1.865, p=0.103), was independently associated with good outcome after adjusting for age and baseline NIHSS score. When adding ONT into the regression model, ONT (OR=0.994, p=0.001), but not EMS with PNP (OR=1.785, p=0.236), was independently associated with good outcome. EMS with PNP, rather than EMS only, improved stroke outcome by shortening ONT. PNP could be a feasible strategy for better stroke care in Chinese urban area.

Stroke is the leading cause of death in China (1). Intravenous thrombolysis (IVT) with recombinant tissue plasminogen activator (rt-PA) can markedly improve clinical outcome in eligible patients with acute ischemic stroke (AIS), yet, the efficacy is strongly time-dependent (2). An urban population-dominated study from Chinese National Stroke Registry reported in 2011, that only approximately 2% of AIS patients received IVT in China, due to the narrow time window (3). More efforts are needed to shorten the time between the onset of stroke symptoms and the initiation of thrombolytic therapy for AIS patients in Chinese urban area.

The use of emergency medical service (EMS) is a potentially important means to improve medical care for AIS. Prehospital notification by EMS personnel can mobilize the resources of the receiving hospital before patient arrival. It has shown that prenotification could provide more timely hospital admission and care for stroke patients, compared to direct arrival to emergency department (ED) without prenotification (4). However, disparities in prehospital infrastructures and care delivery have made it difficult to implement. A study from the northern Italy found that, ED was not notified in 43% patients (466/1084) of acute stroke cases before the arrival of EMS (5). In China, it was reported that only 8.9% AIS patients arrived hospital by choosing EMS (6). Moreover, there is little contemporary city-based data on the association of EMS prenotification with improved timeliness of in-hospital treatment, and even with neurological outcome in AIS patients in Chinese urban area.

Aiming to improve the performance of AIS management, China has initiated the program of stroke center development since January 2015, which stressed the importance of network connection between stroke center and EMS. As one of the qualified comprehensive stroke center located in urban area, we initiated prehospital notification procedure (PNP) since March 2015. The aim of our study was to determine whether prehospital notification by EMS providers in thrombolytic candidates was associated with a reduction in onset to needle time (ONT), and an improvement of neurological outcome after rt-PA treatment.

## MATERIALS AND METHODS

The present study was retrospectively conducted with a prospectively collected stroke registry of a single stroke center. Our hospital is situated in Hangzhou (size: 701.8 km^2^), Southeast China, a typical Chinese urban area, with a densely population of 9 million. Our hospital is a tertiary teaching hospital and comprehensive stroke center that treats about 1.8 thousand patients with acute ischemic stroke (AIS) or transient ischemic attack per year.

Since 2015, we prospectively designed a systemized PNP by cooperating with local EMS system, in an effort to rapidly evaluate and treat AIS patients in our center. The EMS system of Hangzhou was set up in 1992, which belongs to Hangzhou Municipal Health Bureau. All EMS paramedics in Hangzhou city have been trained for early detection and transportation of stroke patients. The decision to transport a patient to a particular hospital or whether to prenotify the hospital was made by individual paramedics based on each patient’s clinical condition.

### Patients Selection

For this study, we enrolled patients who (i) received IVT between March 2015 and March 2016; (ii) had complete follow-up records. In-hospital stroke patients were excluded from this study. Pretreatment demographic, time, clinical and imaging data, comorbid conditions including history of hypertension, diabetes, atrial fibrillation, etc., were prospectively collected in the stroke database by our stroke team. IVT was administered according to the international guidelines (0.9 mg/kg, 90 mg dose at maximum, 10% in a bolus in 1 min with the remaining dose in a 60-min infusion).

### Ethics statement

All subjects had given written informed consent prior to the study, and the protocols had been approved by the local ethics committee. All clinical investigation has been conducted according to the principles expressed in the Declaration of Helsinki.

### Prehospital notification procedure (PNP)

In order to streamline the pre-thrombolysis assessment by eliminating the delays in organizing and transferring patients to image scan after an initial clinical assessment, the PNP allows the whole procedure to be under control by stroke team members in the hospital, who could finish the preparations in advance prior to the formal off-loading of patients in ED. In detail, by using FAST (Face-Arm-Speech-Time) score, paramedics on ambulance would call the stroke team (24-hour shifts) by telephone if a suspected acute stroke patient met any of FAST items when they were still on the ambulance (7). In the phone call, the stroke team would further pick up information about the history of past and present illness, and then pre-notice the ED nurses and neuroimaging technician after excluding the contraindications of intravenous rt-PA. Once arriving, the patient was then immediately transferred to image room after blood drawing by ED nurses.

Patients received IVT but did not prenotice the stroke team were classified as non-PNP group, including those who transferred by EMS (marked as EMS without PNP) and other means of transportation (marked as non-EMS). For non-PNP, the stroke team would be alerted after the emergency neurologist identified a patient as a stroke candidate. Stroke team members would immediately go to meet the patient in emergency room to judge if he is eligible for rt-PA thrombolysis and prenotice neuroimaging technicians to prepare an urgent image assessment for this stroke candidate. Blood drawing would be finished during the process of preparation. After these preparations, stroke team members would transfer this patient to imaging room.

**Figure 1.**
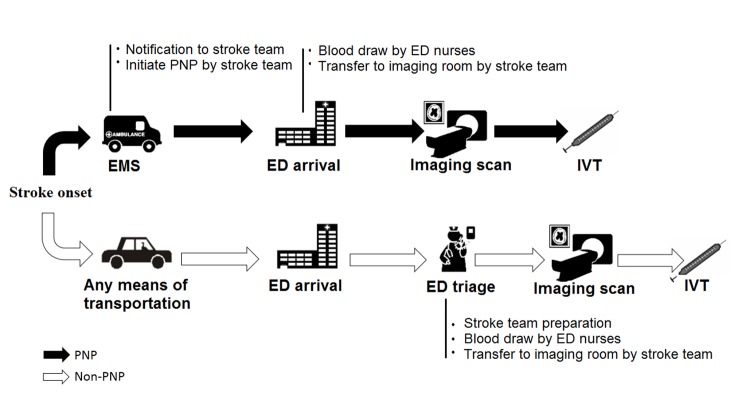
Flow chart of prehospital notification procedures (PNP) and non-PNP. EMS: emergency medical service, ED: emergency department, IVT: intravenous thrombolysis.

From image assessment to IVT bolus, there were no differences in procedures between PNP and non-PNP. These two procedures were shown in Fig. 1. All patients underwent computer tomography (CT) or magnetic resonance imaging (MRI) in accordance with our routine stroke imaging protocol (8).

### Measurements

The start and the end time of each step involved in the procedures was prospectively recorded by using time tracking table. We assessed the time from onset to ED arrival (onset to door time, ODT), ED arrival to imaging time (door to imaging time, DIT), ED arrival to intravenous rt-PA bolus (door to needle time, DNT), and onset to intravenous rt-PA bolus (onset to needle time, ONT). According to the setting of our time tracking table, DNT was mainly comprised of four parts: (i) duration in ED; (ii) ED departure to initiation of imaging scan;(iii) duration of imaging scans; (iv)end of imaging scan to initiation of IVT. Stroke severity was assessed at baseline with National Institutes of Health Stroke Scale (NIHSS). Each table was collected within 24 hours since the end of the procedure and kept by a person specially assigned.

### Outcomes

Hemorrhagic transformation (HT) was identified on 24-hours susceptibility-weighted imaging (SWI) images or CT and classified as hemorrhagic infarction (HI) and parenchymal hemorrhage (PH), according to the European Cooperative Acute Stroke Study (ECASS) definition. Symptomatic hemorrhagic transformation (sHT) was defined as any intracranial hemorrhage associated with an increase of ≥ 4 points of NIHSS, or death (9). Neurological outcome at 3 months was measured by the modified Rankin (mRS) score. Good outcome was defined as 3-month mRS score of 0-2, and poor outcome as score of 3-6.

**Figure 2.**
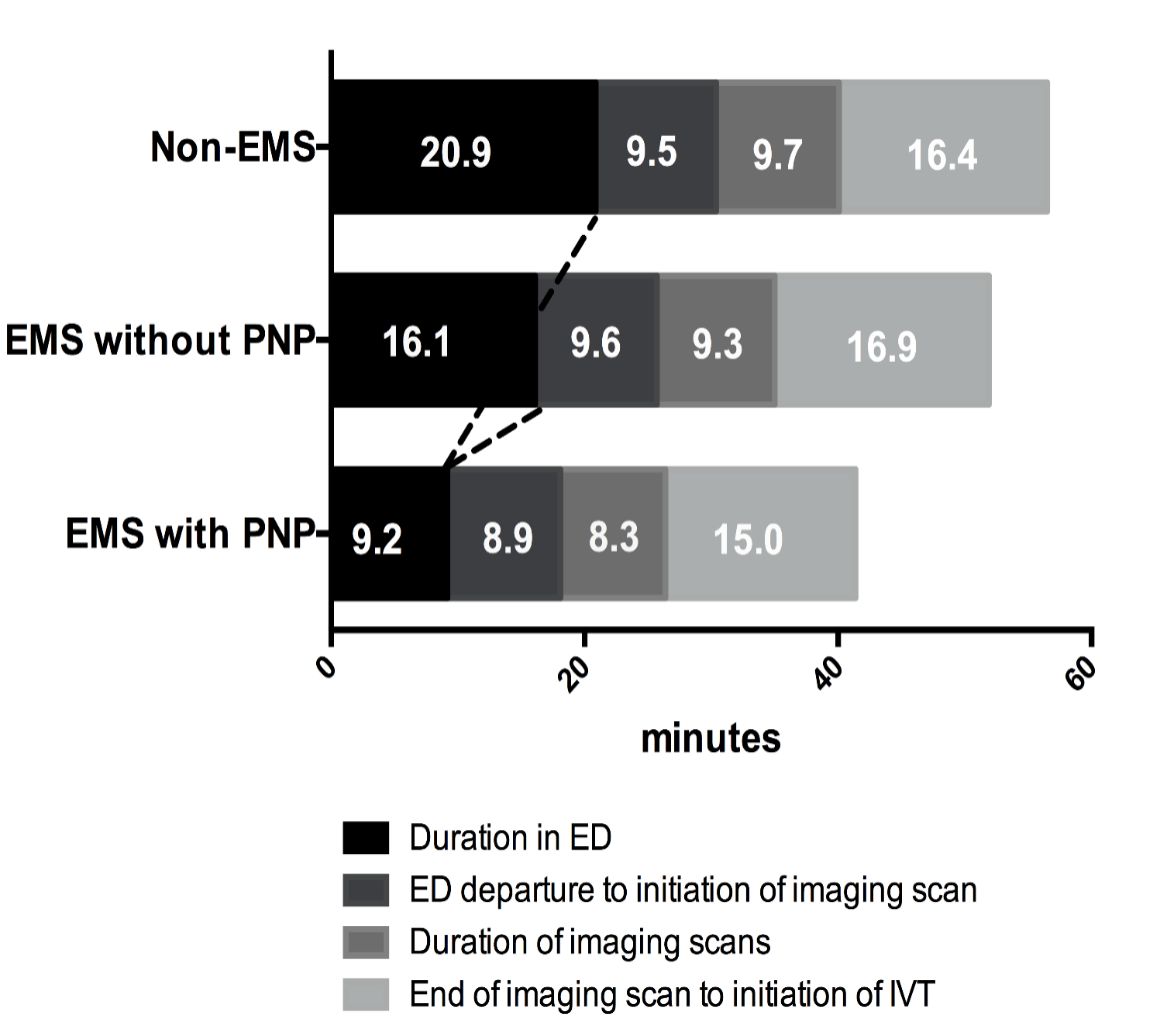
Four-parts durations of DNT in EMS with and without PNP and non-EMS groups. DNT was comprised of four parts: (i) duration in ED; (ii) ED departure to initiation of imaging scan; (iii) duration of imaging scans; (iv) end of imaging scan to initiation of IVT. Significant difference in ED duration part was found between two groups connected by dotted lines. DNT: door-to-needle time, EMS: emergency medical service, ED: emergency department, IVT: intravenous thrombolysis, PNP: prehospital notification procedure.

### Statistical analyses

All metric and normally distributed variables were reported as mean ± standard deviation; non-normally distributed variables as median (25th-75th percentile). Categorical variables were presented as frequency (percentage). Student t test for parametric data or Mann-Whitney U test for nonparametric data was used to compare continuous variables between two groups, whereas Pearson Chi-Square test was used for categorical data. One-way ANOVA or Kruskal-Wallis test was used between multiple groups. The association of PNP and EMS with good outcome were determined by binary logistic regression analysis. Results are reported as odds ratios (OR) with 95% confidence intervals (CIs). A p value of < 0.05 was considered to be statistically significant. All statistical analyses were conducted using SPSS, Version 19.0 (IBM, Armonk, New York).

## RESULTS

### Overall characteristics

For one year, 182 patients were included into analysis. The mean age was 69.2 ± 13.1 years with 65 (35.7%) being female. The median baseline NIHSS was 11.0 (4.8-16.0), the average ONT was 208.8 min and DNT was 52.2 min. Totally, 77 (42.3%) patients were transferred by EMS, of whom 41 (53.2%) patients entered PNP. After IVT, 61(33.5%) patients had HT at 24 hours and 99 (54.4%) patients achieved good outcome

### EMS vs non-EMS

Compared with non-EMS group, patients transferred by EMS (including EMS with and without PNP group) had a higher baseline NIHSS score (median: 13 vs 8, Z = -3.130, p = 0.002), a lower rate of TIA / stroke history (7.8% vs 22.9%, χ^2^ = 7.324, p = 0.007), a shorter ONT (175.1 ± 93.8 min vs 231.3 ± 109.1 min, t = 3.431, p = 0.001), ODT (131.8 ± 86.5 min vs 174.8 ± 105.1 min, t = 2.925, p = 0.004), DNT (46.3 ± 18.7 min vs 56.6 ± 18.3 min, t = 3.713, p<0.001), DIT (21.7 ± 10.4 min vs 30.4 ± 15.7 min, Z= -4.450, p<0.001) and ED duration (12.4 ± 8.4 min vs 21.0 ± 14.4 min, Z= -4.880, *p*<0.001).

In the subgroup of non-PNP patients, EMS without PNP group also showed a higher baseline NIHSS score (Z = -3.049, p=0.002), a lower rate of TIA / stroke history (χ^2^ = 3.324, p=0.046), a shorter ONT (t=2.387, p=0.018) and ODT (t=2.298, p=0.023) in comparison of non-EMS group, but there was no significant difference in DNT between two groups (t=1.206, p=0.230) (see Table 1 and Fig. 2).

**Table 1:** Univariate comparisons among patients transferred by EMS with or without PNP, and other means of transportation (non-EMS).

	Non-EMS n=105	EMS without PNP n=36	EMS with PNP n=41	Test value	p value
Age, y^*^	68.8 ± 12.5	73.4 ± 11.3	66.5 ± 15.4	F=2.810	0.063
Female, n (%)	34 (32.4)	14 (38.9)	17 (41.5)	χ^2^=1.256	0.543
Baseline characteristics					
Smoking, n (%)	42 (40.0)	11 (30.6)	12 (29.3)	χ^2^=1.999	0.368
Hypertension, n (%)	69 (65.7)	21 (58.3)	27 (65.9)	χ^2^=0693	0.707
Diabetes mellitus, n (%) ^&^,^*^	22 (21.0)	3 (8.3)	12 (29.3)	χ^2^=5.246	0.073
Atrial fibrillation, n (%)	38 (36.2)	17 (47.2)	14 (34.1)	χ^2^=1.705	0.426
Hyperlipidemia, n (%)	45 (42.9)	17 (47.2)	14 (34.1)	χ^2^=1.471	0.479
Previous TIA/stroke, n (%) ^&^,^∫^	24 (22.9)	4 (11.1)	2 (4.9)	χ^2^=7.865	0.020
Baseline NIHSS score,IQR^&^	8.0 (4.0-15.0)	14.0 (8.3-18.8)	12.0 (6.5-16.5)	F=4.128	0.018
Baseline SBP, mmHg	153.1 ± 19.9	156.8 ± 29.2	152.5 ± 21.8	F=0.438	0.646
Baseline DBP, mmHg	84.9 ± 11.5	80.4 ± 11.5	83.9 ± 13.9	F=1.855	0.159
Baseline serum glucose, mmol/L^&^	7.8 ± 3.0	6.6 ± 1.6	7.1 ± 2.8	F=2.274	0.106
Receive MRI, n (%)	10 (9.5)	2 (5.6)	0 (0)	χ^2^=4.421	0.110
Time tracking information					
Onset to needle time,min^&^,^∫^	231.3 ± 109.1	182.3 ± 98.1	174.54 ± 93.0	F=5.909	0.003
Onset to door time,min^&^,^∫^	174.8 ± 105.1	130.3 ± 83.4	133.2 ± 90.2	F=4.264	0.016
Door to needle time,min^*^,^∫^	56.6 ± 18.3	51.9 ± 23.8	41.3 ± 10.7	F=10.395	<0.001
Door to imaging time, min^*^,^∫^	30.4 ± 15.7	25.8 ± 12.3	18.1 ± 6.6	F=12.469	<0.001
Duration in ED, min^*^,^∫^	20.9 ± 14.4	16.1 ± 9.5	9.2 ± 5.5	F=14.455	<0.001
ED departure to initiation of imaging scan, min	9.5 ± 5.7	9.6 ± 5.0	8.9 ± 4.0	F=0.233	0.792
Duration of imaging scans, min	9.7 ± 5.4	9.3 ± 4.9	8.3 ± 2.8	F=1.336	0.265
End of imaging scan to initiation of IVT, min	16.4 ± 9.1	16.9 ± 12.6	15.0 ±8.0	F=0.463	0.630
Neurological outcomes					
Good outcome, n (%)	58 (55.2)	15 (41.7)	26 (63.4)	χ^2^=3.726	p=0.155
HT, n (%)	36 (34.3)	11 (30.6)	14 (34.1)	χ^2^=0.177	p=0.915
HI, n (%)	24 (22.9)	6 (16.7)	10 (24.4)	χ^2^=0.781	p=0.941
PH, n (%)	8 (7.6)	3 (8.3)	3 (7.3)	χ^2^=0.781	p=0.941
sHT, n (%)	1 (1.0)	2 (5.6)	1 (2.4)	χ^2^=2.657	p=0.265
Death, n (%)	12 (11.4)	6 (17.1)	3 (7.5)	χ^2^=1.698	p=0.428

* EMS without PNP *vs* EMS with PNP, *p*<0.05

& non-EMS *vs* EMS without PNP, *p*<0.05

∫ non-EMS *vs* EMS with PNP, *p*<0.05

EMS, emergency medical service; PNP, prehospital notification procedure; TIA, transient ischemic attack; SBP, systolic blood pressure; DBP, diastolic blood pressure; INR, international normalized ratio; NIHSS, National Institute of Health Stroke Scale; IVT, intravenous thrombolysis; HT, hemorrhagic transformation; HI, hemorrhagic infarction; PH, parenchymal hemorrhage; sHT, symptomatic hemorrhagic transformation.

**Table 2:** Univariate comparisons between patients with or without good outcome.

	Poor outcome n=83	Good outcome n=99	Test value	p value
Age, y	72.2 ± 12.1	66.6 ± 13.4	t=2.908	0.004
Female, n (%)	31 (37.3)	34 (34.3)	χ^2^=0.178	0.673
Baseline characteristics				
Smoking, n (%)	29 (34.9)	36 (36.4)	χ^2^=0.040	0.842
Hypertension, n (%)	55 (66.3)	62 (62.6)	χ^2^=0.260	0.610
Diabetes, n (%)	14 (16.9)	23 (23.2)	χ^2^=1.129	0.288
Atrial fibrillation, n (%)	35 (42.2)	34 (34.3)	χ^2^=1.174	0.278
Hyperlipidemia, n (%)	32 (38.6)	44 (44.4)	χ^2^=0.644	0.422
TIA/stroke history, n (%)	14 (16.9)	16 (16.2)	χ^2^=0.016	0.898
Baseline NIHSS score, IQR	14.0 (12.0-19.0)	5.0 (3.0-10.0)	Z= -5.923	<0.001
Baseline SBP, mmHg	154.2 ± 21.5	153.4 ± 23.2	t=0.238	0.812
Baseline DBP, mmHg	84.4 ± 11.4	83.3 ± 12.8	t=0.634	0.527
baseline serum glucose, mmol/L	7.6 ± 3.2	7.3 ± 2.5	t=0.686	0.494
Transferred by EMS, n (%)	36 (43.4)	41 (41.4)	χ^2^=0.071	0.790
EMS with PNP, n (%)	15 (18.1)	26 (26.3)	χ^2^=1.735	0.188
Time tracking information				
Onset to needle time, min	228.5 ± 115.9	192.4 ± 95.1	Z=2.146	0.032
Onset to door time, min	174.1 ± 106.6	142.0 ± 91.6	t=2.183	0.030
Door to needle time, min	54.4 ± 21.4	50.4 ± 16.9	t=1.435	0.154
Door to imaging time, min	27.9 ± 15.0	25.8 ± 13.8	t=0.999	0.319
Duration in ED, min	18.4 ± 13.6	16.5 ± 12.3	t=1.006	0.316
ED departure to initiation of imaging scan, min	9.5 ± 5.1	9.3 ± 5.3	t=0.266	0.790
Duration of imaging scans, min	9.8 ± 5.1	8.9 ± 4.6	t=1.121	0.264
End of imaging scan to initiation of IVT, min	16.8 ± 10.1	15.7 ± 9.2	t=0.787	0.433

TIA, transient ischemic attack; SBP, systolic blood pressure; DBP, diastolic blood pressure; INR, international normalized ratio; NIHSS, National Institute of Health Stroke Scale; ED, emergency department; IVT, intravenous thrombolysis; PNP, prehospital notification procedure.

### PNP vs non-PNP

The comparisons between EMS with PNP group and non-EMS group showed similar results to that between EMS without PNP group and non-EMS group. However, there were significant differences in DNT (Z= -4.713, p<0.001), DIT (Z= -5.341, *p*<0.001) and ED duration (Z= -6.216, *p*<0.001) between these two groups.

In the subgroup of patients that transferred by EMS, there were no differences in the rate of TIA/stroke history (4.9% vs 11.1%, χ^2^=1.037, p=0.309), ONT (174.5±93.0 min vs 182.3 ± 98.1 min, t=0.355, p=0.724) and ODT (133.2 ± 90.2 min vs 130.3.4±83.4 min, t= -0.146, p=0.884) between EMS with and without PNP group, whereas EMS with PNP patients were younger (66.5±15.4 y vs 73.4 ± 11.3 y, t=2.211, p=0.030), and had a shorter DNT (41.4 ± 10.8 min vs 51.9 ± 23.8 min, t=2.472, p=0.017), DIT (18.1 ± 6.6 min vs 25.8 ± 12.3 min, Z= -3.188, p=0.001) and ED duration (9.2 ± 5.5 min vs 16.1 ± 9.5 min, Z= -4.122, p<0.001), compared with EMS without PNP patients (see table 1 and Fig. 2).

Since none of patients in EMS with PNP group underwent MRI scan, we did the above comparison after excluding 2 patients who undertook baseline MRI in EMS without PNP group. The results were similar. EMS with PNP patients were younger (66.5 ± 15.4y vs 72.7 ± 11.2y, t=1.975, p=0.052), and had a shorter DNT (41.3 ± 10.7 min vs 50.3 ± 23.5 min, t=2.069, p=0.044), DIT (18.1 ± 6.6 min vs 25.0 ± 12.1 min, Z=-2.908, p=0.004) and duration in ED (9.2 ± 5.5 min vs 15.9 ± 9.8 min, Z= -3.903, p<0.001) than those in EMS without PNP group.

**Table 3:** Multivariate regression analysis for good outcome.

Model 1	OR	95% CI	p value
Age	0.979	0.952-1.007	0.148
Baseline NIHSS	0.812	0.760-0.868	<0.001
TIA / stroke history	1.732	0.628-4.780	0.289
EMS	1.865	0.881-3.946	0.103
**Model 2**	OR	95% CI	p value
Age	0.982	0.954-1.010	0.201
Baseline NIHSS	0.813	0.760-0.869	<0.001
TIA / stroke history	1.790	0.645-4.970	0.264
Non-EMS	-	-	-
EMS without PNP	1.236	0.481-3.177	0.661
EMS with PNP	2.613	1.062-6.427	0.036
**Model 3**	OR	95% CI	p value
Age	0.972	0.943-1.001	0.058
Baseline NIHSS score	0.818	0.765-0.874	<0.001
TIA / stroke history	1.294	0.444-3.767	0.636
Non-EMS	-	-	-
EMS without PNP	0.897	0.326-2.469	0.834
EMS with PNP	1.785	0.684-4.653	0.236
Onset to needle time	0.994	0.990-0.998	0.001

PNP, prehospital notification procedure.

### The association of EMS and PNP with good outcome

Univariate comparison showed that, there were no differences in the rate of good outcome, sHT and death among three groups (all *p*>0.05, see Table 1). However, patients in EMS with PNP group had a trendy higher rate of good outcome in comparison of EMS without PNP group (63.4% vs 41.7%, χ^2^=3.642, p=0.056).

As shown in Table 2, patients with good outcome were younger, had lower baseline NIHSS score, shorter ONT and ODT, compared with those with poor outcome (all *p*<0.05). After adjustment for age, baseline NIHSS score and TIA / stroke history, multivariate regression analysis showed that EMS was not associated with outcome (OR=1.865, 95% CI=0.881-3.946, p=0.103), and EMS without PNP group showed no better outcome than non-EMS group (OR=1.236, 95% CI=0.481-3.177, p=0.661). However, EMS with PNP (OR=2.613, 95% CI=1.062-6.427, p=0.036) was independently associated with good outcome in comparison of non-EMS group. After adding ONT into the regression analysis, only ONT (OR=0.994, 95% CI=0.990-0.998, p=0.001) and baseline NIHSS (OR=0.818, 95% CI=0.765-0.874, *p*<0.001), but not EMS with PNP (OR=1.785, 95% CI=0.684-4.653, p=0.236), were independently associated with good outcome (Table 3).

## DISCUSSION

During one-year period, 42% patients were transferred by EMS and about half of them arrived at our stroke center via PNP. By the implementation of PNP, not only the ODT was reduced, but also the whole in-hospital treatment procedure was greatly speeded up, especially for the time in ED. Therefore, the ONT was visibly shortened, accompanied with better neurological outcome.

In the current study, our PNP effectively reduced the in-hospital delay, indicating that stroke patients can receive more timely treatment by activating PNP with prenotification (10). However, this result was inconsistent with a retrospective analysis from “Get with the guideline”, which found that prenotification by EMS was not effective enough to reduce DNT. This negative finding might be attributed to the phenomenon that EMS prenotification was more an EMS strategy than hospital strategy. Hospital respondents might just report what they should do or plan to be doing, as opposed to actual practice after receiving prenotification (11). In our center, the effectiveness of PNP was probably due to the activation of stroke team who would finish the preparations in advance, but not await to do after patients’ arrival. The philosophy behind this reduced DNT is, ‘to do as little as possible after the patient has been arrived in the clinic, and as much as possible when the patient is on the way to the clinic’ (12).

Due to the reduction of in-hospital delay, we found that PNP, rather than EMS, was associated with the improvement of neurological outcome. It is supported by a previous finding that improved timeliness of IVT was associated with the improvement in clinical outcome (13). However, a study from Busan metropolitan area of South Korea showed that EMS prenotification effectively was unable to improve 3-month neurological outcome, even though the in-hospital processing times was reduced (non-prenotification vs. prenotification: 47.7 min vs. 28.9 min) (14). This was attributed to the fact that ONT was not reduced by prenotification (without notification vs. with notification: 122.6 min vs. 150.4 min). In that study, most patients in the prenotified group stopped at other hospitals which were not able to provide IVT, while most patients in the non-prenotified group resided relatively near their stroke center. Therefore, there have been a huge prehospital time loss in the prenotified group than that in the non-prenotified group. Different from that, patients of PNP group in our study showed a significantly shorter ODT than non-PNP group. It may be attributed to that EMS paramedics directly prenotify stroke team member and transferred patients rapidly to our center without any stop at other hospitals as they were trained before the initiation of PNP in our hospital. The other study from Spain reported that the Stroke Code (SC) which involved the prenotification system in the North Barcelona could influence the access to thrombolysis and outcome. However, the effectiveness degree of SC prenotification was calculated by comparing with patients who were transferred from hospitals that could not provide thrombolytic therapy (15). Therefore, to our knowledge, current study is the first to clearly demonstrate that prehospital notification was an effective method to improve stroke outcome.

Actually, ODT in PNP group was also reduced due to the use of EMS, which is consistent with the findings that patients transferred via EMS was associated with fast hospital arrival (16). However, our rate of EMS utilization was relatively low (42.3%) in comparison of other centers (56.1%-80.3%) (17, 18), reflecting a serious phenomenon in China that the public had poor knowledge about emergency stroke care. Of note, from our data, we found that patients with history of TIA/stroke were more unlikely to choose EMS to access to our stroke center after the recurrence of stroke. This phenomenon was also found in a previous investigation where it was explained that their relatives or patients themselves had bad experience with EMS (19). Although further investigations are still needed to reveal actual causes for this phenomenon, it may highlight the importance of the education for public awareness of emergency stroke care, considering that patients with previous TIA/stroke knew their illness well, but should wait for EMS arrival as it provides a faster access to IVT than other means of transportation.

Compared with other stroke centers using EMS prenotification (14, 20), we took only one year, since the implementation of PNP, to shorten the in-hospital delay from 55 min to 41 min. In addition, the rate of PNP was up to 50%, which is equal to or even higher than that of other centers, indicating that PNP is probably much easier to perform in China. Additionally, as a typical Chinese urban, our city is a densely-populated region, spanning only 3068 square miles with 6.6 million residents. Therefore, our stroke patients are likely to arrive in hospital more rapidly than those in rural area due to short distance to a qualified hospital. By shortening in-hospital delay on the basis of the reduced time to hospital with EMS, PNP would be an ideal method to be applied in Chinese urban area where there is a high demand for stroke care.

Limitations in this study include a small sample size and potential selection bias because our study was a short-term survey and only focused on patients who received IVT, we do not know the effect of prehospital notification on patients who did not receive IVT. A long-term observation and further validation in all the patients suspected of stroke and transferred by EMS are needed. Regardless, our results are intriguing and support current guideline recommendations that advocate for advanced hospital notice of inbound stroke patients by EMS, and PNP was an effective strategy when a close collaboration was developed between well-trained and organized EMS systems and stroke centers.

In conclusion, PNP significantly improved stroke outcome by shortening the time from stroke onset to treatment. PNP is a feasible strategy for better stroke care in Chinese urban area.
